# Satisfaction of Township Hospitals Health Workers on How They Are Paid in China

**DOI:** 10.3390/ijerph182211978

**Published:** 2021-11-15

**Authors:** Beibei Yuan, Yahang Yu, Hongni Zhang, Huiwen Li, Chen Kong, Wei Zhang

**Affiliations:** 1 China Center for Health Development Studies, Peking University, 38 Xue Yuan Road, Haidian District, Beijing 100191, China; 2 Department of Global Health, School of Public Health, Peking University, 38 Xue Yuan Road, Haidian District, Beijing 100191, China; 1911210142@bjmu.edu.cn (Y.Y.); 2011210109@stu.pku.edu.cn (C.K.); 1610306226@pku.edu.cn (W.Z.); 3 School of College Industry & Commerce, Shandong Management University, 3500 Dingxiang Road, Changqing District, Jinan 250357, China; 14438120130335@sdmu.edu.cn; 4 China Population and Development Research Center, Beijing 100191, China; hli@bjmu.edu.cn

**Keywords:** job satisfaction, payment, primary health workers

## Abstract

Background: Township Hospitals (THs) are crucial providers in China’s primary health delivery system. Low job satisfaction of THs health workers has been one of biggest challenges to strengthening the health system in China. Even huge amounts of studies confirmed low remuneration level as a key demotivating factor though few studies have explored the feelings of health workers on how they were paid. Objective: To analyze how the key design of Performance-based Salary System (PBS) influences the satisfaction of health workers on the payment system in China’s THs. Method: A cross-sectional study was conducted in 47 THs in Shandong China, and a total of 1136 participants were recruited. Expectancy theory was applied to design the measurements on designs of PBS. The associations between PBS design and satisfaction of health workers were analyzed by logistic regression. Results: Three key components of PBS design were all related to the satisfaction of health workers. Those health workers who were aware of assessment methods were more likely to be satisfied with how they were paid (OR = 2.44, *p* < 0.001) compared with those being not aware of the methods. The knowledge on personal performance was also associated with being satisfied (OR = 3.34, *p* < 0.001). The percentage of floating income in total income was negatively associated with the satisfaction, and one percentage point increase in floating income proportion could result in the possibility of being satisfied decreasing by 2.82% (95%CI −4.9 to −0.7, *p* = 0.01). Subgroup analysis found that only in those with lower value on monetary income, the negative influence of more floating income was significant. Conclusions: When policymakers or managers apply performance-related payment to incentivize certain work behavior, they should pay attention to the design details, including keeping transparency in the performance assessment criteria, clear performance feedback, and setting the proportion of the performance-related part based on the preference of health workers in certain cultural settings.

## 1. Background

China’s health delivery system has a hierarchical system corresponding with administrative levels, consisting of health providers in national, provincial, prefecture, county, township (rural areas)/community (urban areas), and village levels. Township hospitals (THs) are taking a pivotal role in the three-tier delivery system of rural areas, connecting the hospitals and village clinics. County and higher hospitals are in charge of providing specialized medical services for the covered population and providing technical supports to medical services provision to lower level hospitals and THs. THs also take responsibility in provision of medical services for common diseases and training and supervising the village clinics (the lowest level of healthcare delivery system) in providing first contact services to rural residents. The significant role of THs is also reflected in the THs having a dual function in providing the rural populations with both basic medical services (clinical treatment for diseases) and public health services (preventive care, health management services, and health information collection and management). THs and village clinics are primary healthcare providers in China. THs’ good operation plays a key role in the performance of the whole health system. However, THs continue to have a disadvantaged development status, especially compared with the development of county and higher hospitals in China without a compulsory gate-keeping system. The direct expression is patients bypassing THs and seeking health care in higher hospitals even for minor disease conditions [1].

The reforms to strengthen the primary health system started in 2009, when China launched the health system reform. There are three major policies targeting THS. The first one is political commitments being ensured and public financial supports being increased to strengthen the infrastructure and capacity of health workers in THs [2]. The second is eliminating mark-ups on drugs dispensed by THs with aims to control their revenue-pursuing behaviors; at the same time, local governments needed to increase the budgets to balance THs’ financial loss from drugs benefits; however, this part of financial support is dependent on the local government’s financial capacity and cannot make up THs’ drug revenue loss in most cases [3]. Another crucial policy is “equalization of basic public health services”, in which governments subsidize the THs based on the number of covered residents for providing the essential public health services package [4]. With these reforms, infrastructure, financing level, and revenue sources, services package of THs experienced significant improvement. However, the relative disadvantaged role of THs seems unchanged: the proportion of healthcare services provided by primary care decreased by 7%, and the bed occupation rate of THs was 56.3% compared with bed occupation rates of higher hospitals, at 83.6% in 2019 [2].

More and more studies and analysis on the health system in China agree that lack of qualified and motivated health workers was the bottleneck impeding THs development [1,2,5]. Job satisfaction of THs health workers, an affective indictor of work motivation, stayed at a moderate or low level [3]. As the consequences of low motivation, the difficulty in attracting medical graduates to THs persists; turnover intention of existing THs health workers is higher than that in health workers of higher level hospitals [6], and those health workers really leaving THs were more likely to be those with higher professional title level and longer working experience [7]. Another common finding from existing evidences is that the income and remuneration from work is still the most prominent factor causing job dissatisfaction in China [8].

The impacts of financial rewards on job satisfaction are actualized two ways: overall remuneration level and how to pay the remuneration. Under the current operation status of THs in China, the health workers of THs are employees of THs. The pay level of health workers in THs was only about 30–50% of their expected pay level [5]. Regarding the payment method to health workers in THs, it is a kind of performance-based salary (PBS) based on policy documents issued by national health authority. Total income of salary includes two parts: one basic salary (60–70% of the total income) and one performance-based bonus salary (30–40% of the total income) [5]. With the long-term challenges in low motivation, recently, the policies have started to prioritize the refinement of salary payment methods and try to use financial incentives to motivate THs health workers, so policies in recent years have mentioned “allowance to increase the total performance salary level for health workers in THs”, “enlarge the variation of total income distribution among THs employees”, and “appropriately increase the proportion of performance-based bonus salary in total salary” [5]. In practice, because THs facility has the autonomy in setting the proportion of the performance-based part in total salary, the specific design of PBS has varied in different areas and different THs in the same area. However, not like the huge amount of studies confirming remuneration level as one important demotivating factor, few published studies have explored the detailed design of PBS in THs and whether THs health workers were satisfied with current design of PBS.

As salary payment reform has become the priority in policy agenda to motivate THs health workers in China, it is necessary to research how to improve the design of payment. Based on motivation theories, the mechanism of the payment works as an effective payment system that can keep the health workers motivated, i.e., a harmony status between internal driver (factors individual needs) and external conditions (factors organizations can offer). This harmony status is expressed in behaviors, such as health workers being willing to exert effort to performance goals desired by the organization, and at the same time is expressed in health workers feeling satisfied [9]. Therefore, both behavior and feeling of health workers can be criteria to judge the effectiveness of the payment system.

Globally, there are many evidences on Performance-based Payment (PBP) targeting individual health professionals. Several high-quality systematic reviews [10,11] have synthesized the effects of PBP on behaviors of individual health providers. High heterogeneity in findings have reminded the researchers that the health system context, health organization settings, and different designs of PBP may explain these differences. Regarding the design of PBP, some researchers [12,13] have also summarized the hypothesized factors affecting the size of effects of PBP, including type of performance measures, type of performance target, size of incentive, frequency of monitoring and feedback, and frequency of payment. However, how the health workers perceive and feel the PBP has been seldom studied. Some qualitative studies [14,15] found the negative consequences of PBP on feelings of health workers because PBP using financial incentives to change the medical professional behaviors is against medical professionalism and clinical autonomy.

Without conclusion with certainty from global evidences on the impacts of PBP and with the special setting in China’s health system, it is necessary to analyze the design of PBS in THs of rural China and examine how the design of PBS influences the satisfaction of health workers with the intention to provide policy suggestions on how to improve the design of PBS and the motivation and performance of health workers in primary healthcare facilities.

**Figure 1:**
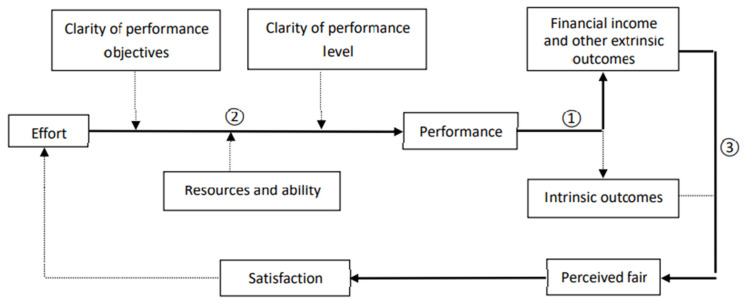
The framework of this study based on expectancy theory.

To achieve the above study objective, the design of this study and selection of PBS design characteristics was based on a classic motivation theory—expectancy theory [9] (Figure 1). This theory hypothesizes that it is the individual “perceived” link between outcomes he/she will get with his/her work outputs guiding his/her work behaviors and feeling of fairness. The outcomes used for motivating health workers are usually the financial rewards, which are valued in the utility function of most of human beings based on traditional economy theory. If, under a certain payment system design, health workers perceive that financial rewards come anyway or do not come regardless of their effort level and work outputs, or if health workers do not value extra financial rewards, then this payment system will not work for changing work morale and behaviors. This theory emphasizes that the link between the financial rewards and work outputs must be realized and believed by health workers. For strengthening this belief on the link, PBS design needs to ensure that all workers are aware of the amount of financial rewards being followed from the explicit contents and levels of work performance (Link ①, Figure 1), all people concerned are aware of their performance (Link ②, Figure 1), and the amount financial rewards linked with the performance are meaningful compared with their total income (Link ③, Figure 1). All these designs work only if health workers value the extra financial rewards.

## 2. Method

Based on the above theory and hypothesis, a cross-sectional survey study was designed.

### 2.1. Data Collection

We conducted a cross-sectional study in three countries of Shandong province. Located in eastern China, Shandong Province is the second largest province in terms of the population and the third ranking province in terms of GDP per capita per year. Three counties, including Shouguang, Huantai, and Yanggu, were randomly selected to represent the province’s high-, middle-, and low-level economic region. All the THs in these three counties were selected, with 16 from Shouguang, 13 from Huantai, and 18 from Yanggu. All the primary health workers on duty on the investigation day were asked to participate in the structured questionnaire survey, and the health workers were invited to a meeting room and filled out the self-administrated questionnaire with research staffs available to address their questions. Finally, a total of 1139 participants were recruited. The study has been approved by the Ethics Review Board of the School of Public Health, Peking University. Informed consent was obtained from all participants prior to questionnaire administration.

### 2.2. Measures and Variables

Based on the above theory, this study hypothesizes that a well-designed PBS strengthening the link between rewards and outputs can help health workers built clear expectation on their behavior direction, efforts, and corresponding rewards and will be more satisfying for health workers.

We developed three questions measuring health workers’ perception on PBS design following the above three principles, including “awareness of the performance assessment criteria”, “awareness of your own performance”, and “the perceived proportion of performance-based salary to total income”, and the answers to three questions are the key independent variables. The content validity of three questions were tested through several discussions with researchers in the area of work motivation to ensure accuracy, readability, and clarity of questions. The construct validity test is not applicable since each aspect of perception on PBS was assessed with one question directly instead of in the form of a scale with various questions.

The dependent variable, satisfaction of health workers on PBS, was measured on a 5-point Likert scale from 1 (very dissatisfied) to 5 (very satisfied); the 5-point Likert scale was collapsed into a binary category of dissatisfied (very dissatisfied, dissatisfied, moderate) and satisfied (satisfied, very satisfied) in analysis.

We also controlled some other factors potentially influencing the satisfaction on PBS. Firstly, we used “financial income is one of three top motivators for me” (yes, no) to measure level of health workers’ preference on monetary rewards, as the theory basis assumed the extent of value on monetary income could influence whether PBS design works in changing satisfaction and behavior of health workers. We also controlled some factors potentially influencing the satisfaction on PBS design, including the satisfaction on total income level (satisfied, very satisfied), gender (female, male), age (<25, 25–35, 35–45, 45–55, ≥55), educational background (high school or below, junior college, bachelor’s and above), and some work-related covariates, including professional status (lower than primary, primary, intermediate, senior/deputy senior), employment status (permanent, temporary), type of primary healthcare workers (physician, nurse, public health worker), and dual work (yes, no).

### 2.3. Patient and Public Involvement

There is no patient involved in the study.

### 2.4. Statistical Analysis

The total participants were divided into three subgroups according to the types of job (Figure 1) for sample description and some comparison among health workers with different types of jobs. THs health workers were classified into physicians, nurses, and public health workers (PHWs) according to the department where they are working (clinical department or public health department). Descriptive statistics were used to describe the characteristics of the THs health workers with different types of jobs. Chi-square tests were conducted to determine the differences among three types of health workers.

Then we applied multivariate logistic regression to identify the influencing factors of satisfaction on PBS, especially whether “awareness of the performance assessment criteria”, “awareness of your own performance”, and “the perceived proportion of performance-based salary to total income” affected satisfaction on PBS. The participants were split into two groups by satisfaction on income (dissatisfied and satisfied), and we conducted multivariate logistic regression for two subgroups to identify the association between satisfaction with total income level and satisfaction with PBS design. The odds ratios (ORs) and their corresponding 95% confidence intervals (CI) were reported. All comparisons were 2-sided and were considered statistically significant as *p* < 0.05. The quantitative data was analyzed using Stata14, and missing data were omitted.

## 3. Result

### 3.1. Basic Characteristics of Participants and PBS Design in THs

Table 1 shows the characteristics of participants. This study consisted of 1136 health workers in THs, including 43.49% (*n* = 494) physicians working in clinical department and mainly providing inpatient and outpatient services, 24.47% (*n* = 278) nurses, and 32.31% (*n* = 367) public health workers working in public health department and mainly providing preventive and management care. Compared with physicians (19.27%, *n* = 95) and nurses (10.21%, *n* = 28), a higher percentage of public health workers was in the group aged higher than 45 (21.37%, *n* = 78). Physicians had a higher education background, with 48.79% (*n* = 436) obtaining a bachelor’s degree or higher, and public health workers had lowest education background, with 29.43% (*n* = 108) only graduating from higher middle school. In the investigated physicians, a higher proportion of them was permanently employed (87.65%, *n* = 433), which implied their salaries were mainly from government financing; a lower proportion of nurses (64.03, *n* = 178) and PHWs (69.21%, *n* = 254) was permanently employed by THs, which implies nurses and PHWs were more likely to be paid by earnings of THs.

The investigated health workers also had their subjective judgement on the design of PBS based on their experiences. We found that the median percentage of floating income to total income is 16.67%, which means, on average, 16.67% of total income is believed by health workers to be reliant on their performance. Physicians (16.67%) and nurses (17.00%) had a similar subjective prediction on the level of performance-based income proportion, but in the opinion of PHWs, the percentage of floating income was relatively lower (11.63%). The majority of health workers (75.18%) were aware of performance assessment methods, i.e., the performance requirements of managers. Most of investigated health workers (79.86%) were also aware of their performance level as evaluated by managers.

Three kinds of health workers did not differ in their value on financial income, with on average half (49.87%) of them rating income as the factor they valued most compared with other factors, which included training, promotion, infrastructure, and equipment in work place, etc. Only 21.72% of health workers were satisfied with the total income level they earned from current work. Nearly half of them (43.87%) are satisfied with the PBS design, and PHWs (52.51%) had higher level of satisfaction on PBS design than physicians (39.34%) and nurses (40.52%).

### 3.2. The Influencing Factors of Satisfaction with Salary Payment Methods

Table 2 shows results of logistic regression in the total sample and provides the association between PBS design characteristics and health workers’ salary payment methods, controlling basic personal/job characteristics of health workers.

The work role was associated with the satisfaction level. Compared with physicians in charge of clinical services provision, nurses were (OR = 1.76, *p* = 0.01) more likely to be satisfied with how they were paid, and PHWs were (OR = 1.94, *p* < 0.001) more likely to be satisfied with existing PBS.

The design of PBS could influence the satisfaction status of health workers. The percentage of floating income in total income was negatively associated with the satisfaction, and one percentage point increase in floating income proportion could result in the possibility of feeling satisfaction decreased by 2.82% (95%CI −4.9 to −0.7, *p* = 0.01). Those health workers who were aware of assessment methods were more likely to be satisfied with how they were paid (OR = 2.44, *p* < 0.001) compared with those who were not aware of the assessment criteria and targets. The knowledge of personal performance was also associated with being satisfied with the payment methods (OR = 3.34, *p* < 0.001).

### 3.3. Different Influencing Factors for Health Workers with Different Value on Financial Income

The impact of PBS design on satisfaction were different for the health workers with different preferences on monetary income. As Table 3 shows, in health workers with higher value on monetary income, those having awareness of the assessment methods (46.32% being satisfied vs. 14.01% being satisfied, *p* < 0.001) were more likely to be satisfied with the payment methods; those having awareness of their own performance (46.51% being satisfied vs. 11.20% being satisfied, *p* < 0.001) were more likely to be satisfied with the payment methods. For those health workers with lower value on monetary income, the impact of PBS design on satisfaction became weak. As Table 3 shows, in those with higher value on monetary income, the associations between awareness of assessment methods/performance and satisfaction were not significant statistically. However, in those with lower value on monetary income, the negative association between proportion of floating income to total income and satisfaction status became significant, and higher proportion of floating income was associated with lower possibility of feeling satisfied (β = −5.00, *p* = 0.04).

## 4. Discussion

Based on the expectation theory, in this study, we hypothesized that a satisfying PBS design should strengthen the link between rewards and outputs, increase the awareness of health workers on their work outputs, and balance an appropriate proportion of performance-linked rewards with the total income. The analysis on data from three counties in Shandong Province of China found that the health workers who were aware of assessment methods and had knowledge on personal performance were linked with greater satisfaction with PBS, and the increase in floating income proportion was linked with decreased possibility of feeling satisfaction. The analysis also found that the influence of being aware of assessment and performance on satisfaction was only significant in those health workers with strong preference for financial rewards, and the negative influence of increasing proportion of floating income was only found in those health workers with lower preference for financial rewards.

Being aware of assessment methods was found to be positively related to satisfaction of health workers in THCs in this study. Theoretically, being clear on the performance assessment criteria can build the perceived link between different levels of their performance and different levels of income they can earn, which is the basis for satisfying their needs for the monetary rewards. International evidences have shown that financial incentive to reward better performance is one of most effective measures to improve the morale and change the behavior of health workers [16]. Fu’s study collecting data from three provinces in China also found that a self-reported link between income and performance assessment was related to satisfaction of health workers with the payment methods [17]. Some findings support that the linkage between promotion and performance can also increase the work satisfaction in China, and the promotion in professional title in PHIs directly brought the income increase [18].

This study also found that having knowledge of personal performance was linked to the satisfaction of health workers with PBS. Job feedback through different methods, including the feedback of manager, patients, and public performance reporting, has been proven as an important motivator for health workers [19,20,21]. Knowing the performance level can help health workers understand the gap to target and judge the fairness of payment and then help to improve the performance if feedback is combined with supportive suggestions. Some other concepts of management methods with similar practices in helping employees be aware of their performance, such as task-oriented leadership [22] and supportive leadership [23], were also found to be factors influencing job satisfaction in health sectors. A study in China also obtained the same findings: the performance feedback and public reporting were both correlated with satisfaction of health workers [17].

It was found that the percentage of floating income in total income was negatively associated with the satisfaction. One possible reason is that the data of this study is from Shandong province, where the Confucius culture was born, relatively following traditional Chinese culture strongly; therefore, collectivism, the doctrine of the mean, and preference for stability are more dominant in the culture of the workplace [24]. Hence, health workers are more likely to have positive feelings about less fluctuating and less competitive payment methods. Even from a global perspective, feelings of security within a job are preferred by employees and are listed as key contributors to job satisfaction, with security being one facet in many job satisfaction scales [25,26]. Existing studies have analyzed the relationship between proportion of performance-based income and the behaviors of health workers and have varied findings with different settings and heterogeneity in specific payment design [10,11]. Some studies concluded that low proportion of income being linked to performance was not enough to change the behavior of health workers [27,28], and a small gap in income levels among health workers with difference performance could not incentivize the performance improvement [29]. One study used data in PHIs health workers in China and found that higher level of performance-based rewards was related to better control of blood pressure for the contracted patients [30]. Regarding the influence of linking income with performance on the feelings of health workers, theoretical analysis based on self-determinant theory explains that too much financial incentives could destroy intrinsic motivation and reduce the enjoyment of health workers of tasks in the long term [31,32]. Some qualitative studies found that pay-for-performance directly using financial incentive to guide the behavior of health workers was detrimental to the autonomy and professionalism of health professionals [14,15]. Though bonding income with performance could push health workers to work harder on the incentivized targets, as earning more income is a driver of health workers to make more efforts, the fatigue of efforts and pressure also decrease the utility of health workers at the same time [33]. Thus, each individual health worker would determine the direction of their behavior and efforts level to balance the utility increase from expected income and utility decrease from fatigue of efforts [34], and in the balance process, those with less value on income would have lower willingness to make efforts toward the performance goal. Similar to what we found in this study, the relationship between higher proportion of performance-based income and lower satisfaction was more significant in those health workers with lower preference for monetary rewards, which meant linking the proportion of income with performance to too great a degree was not welcomed by health workers with less value in monetary income.

In this study, it was also found that work role was related to the satisfaction with PBS, especially for public health workers who were more satisfied with PBS design compared with doctors. In China, “Equalization of Basic Public Health Services” (EBPHS) policy defines a basic public health services package. In THs, public health workers are mainly in charge of EBPHS tasks. Policy EBPHS strongly emphasizes the need to track performance and has designed explicit performance targets to ensure the uniform enforcement of the service packages [4]. Accordingly, in the facility level, the performance assessment criteria for individual public health workers is also designed according to the requirement of policy on EBPHS package, and the performance criteria usually is the rewards for each instance of public health service provision, like 15 Yuan RMB for each diabetes patient follow-up management [35]. In addition to clear performance assessment methods, EBPHS funds are all from government subsidies and are more stable and predictable once the performance targets are achieved. In contrast, the revenues from providing medical services, the main resources to pay doctors and nurses, are more dependent on quantity of clinical services delivered. Therefore, the income of doctors is closely related to the volume of patients, which implies higher level of instability for doctors’ income level. In summary, higher levels of satisfaction of public health workers on PBS are due to clear performance assessment methods and less fluctuation in income level, which are consistent with the above findings on relationship between several PBS design characteristics and the satisfaction of PHWs on PBS.

This study used satisfaction with PBS as dependent variable but not overall job satisfaction. This choice is based on the following considerations. Firstly, there are two commonly applied measurements for job satisfaction: a global satisfaction or satisfaction with various aspects of job [36,37]. Compared with measurement of overall satisfaction, assessing satisfaction on different job components had the advantage of obtaining deeper insight into the specific management or environment factor and directing more targeting interventions to motivate employees [38]. Secondly, health workers’ satisfaction level with how they are paid is an important contributor to overall job satisfaction. A systematic review on determinants of satisfaction in urban community health workers found that health workers’ perception on income distribution, performance assessment, and management were important contributors [39]. A review also found that nurses perceiving supervisors’ support in performance assessment was an associated factor of job satisfaction [40]. The role of satisfaction on payment methods in overall job satisfaction can also be reflected by the facets covered by existing job satisfaction measurement scales. Though not all scales cover “payment methods” per se as a dimension, the meanings of “payment methods” are actually measured in other dimensions with different perspectives. For example, in Minnesota Satisfaction Questionnaire [26], one of the most frequently used scales, “My pay and the amount of work I do” and “The praise I get for doing a good job” are related to fairness of payment and performance feedback. A study on THs of western China used a self-developed job satisfaction scale covering “reward distribution” in the “job reward” dimension [41]. Lastly, though we used feelings but not behavior indicators to judge the design of PBS, satisfaction has been proven as one of the greatest predictor of mobility, productivity, and service quality of health workers [9]. Based on motivation theories, for those employees who are really motivated, being satisfied should happen together with behaving as performance requirements. Therefore, identifying factors relating to dissatisfaction with payment methods and proposed suggestions based on these relationships could directly contribute to the overall job satisfaction and then potentially push performance improvement.

Several limitations of our study should be mentioned. First, this study used the cross-sectional data and could only find the correlation between PBS design and satisfaction of health workers, and we cannot draw any causal inference from our findings. The results should not be interpreted as the effect of the PBS design characteristics on satisfaction of health workers. Second, the measurements of perception of health workers of design of PBS were designed by a research team because there are no validated scales on the evaluation of performance-based payment design. Lastly, this study explores the relationship between PBS design and feelings of health workers, and it will provide more complete evidence on better payment design if future studies add the influence of PBS design on work behavior. Nevertheless, this study only analyzes the feelings of health workers on payment design, and this has been sufficiently useful for some suggestions on improving the welfare of health workers and sustainability in preserving the influence of payment methods on performance. In addition, separate analysis on satisfaction may remind that PBS may have a possible opposite influence on feelings compared to its influence on performance. Though linking more income with performance may stimulate the behavior change in a certain time period, this impact may not be sustainable if the design cannot improve the satisfaction of health workers in the existing payment system and work environment.

## 5. Conclusions

Based on survey data from three counties of Shandong Province in China, this study found that clarifying the link between rewards and performance targets, increasing the awareness of health workers on their performance, and setting an appropriate proportion of performance-linked rewards in the total income were also related to primary health workers being satisfied with the design of their payment methods. If policymakers or managers of primary health institutions are using the performance-related payment methods to incentivize certain work behavior, and they should pay attention to some design details, including keeping transparency in the performance assessment criteria and levels of rewards for different performance, implementing feedback of performance to each health worker, and setting the proportion of performance-related income based on the preference of health workers in certain cultural setting, with awareness that a highly competitive payment system may not be acceptable to all cultures and work places.

## Figures and Tables

**Table 1:** Characteristics of investigated health workers in THs.

Characteristics	Overall *n* = 1139, *n* (%)	Physicians *n* = 494, *n* (%)	Nurses *n* = 278, *n* (%)	Public Health Workers *n* = 367, *n* (%)	*p*
Gender					
Male	397 (34.95%)	267 (54.16%)	2 (0.72%)	128 (34.97%)	<0.001
Female	739 (65.05%)	226 (45.84%)	275 (99.28%)	238 (65.03%)	
Age					
<25	53 (4.68%)	16 (3.25%)	21 (7.66%)	16 (4.38%)	<0.001
25–34	335 (29.59%)	118 (23.94%)	95 (34.67%)	122 (33.42%)	
35–44	543 (47.97%)	264 (53.55%)	130 (47.45%)	149 (40.82%)	
45–54	155 (13.69%)	66 (13.39%)	27 (9.85%)	62 (16.99%)	
≥55	46 (4.06%)	29 (5.88%)	1 (0.36%)	16 (4.38%)	
Educational Background					
Bachelor’s and above	436 (38.28%)	241 (48.79%)	104 (37.41%)	91 (24.80%)	<0.001
Junior college	479 (42.05%)	178 (36.03%)	133 (47.84%)	168 (45.78%)	
High school or below	224 (19.67%)	75 (15.18%)	41 (14.75%)	108 (29.43%)	
Professional title					
Senior/deputy senior	17 (1.50%)	14 (2.85%)	0 (0.00%)	3 (0.82%)	<0.001
Intermediate	394 (34.77%)	203 (41.26%)	103 (37.18%)	88 (24.18%)	
Primary	504 (44.48%)	223 (45.33%)	130 (46.93%)	151 (41.48%)	
Lower than primary	218 (19.24%)	52 (10.57%)	44 (15.88%)	122 (33.52%)	
Employment status					
Temporary	274 (24.06%)	61 (12.35%)	100 (35.97%)	113 (30.79%)	
Permanent	865 (75.94%)	433 (87.65%)	178 (64.03%)	254 (69.21%)	<0.001
Percentage of floating income					
Median	16.67%	16.67%	17.00%	11.63%	<0.001
<15%	428 (37.58%)	167 (33.81%)	93 (33.45%)	168 (45.78%)	<0.001
15–29.99%	398 (34.94%)	192 (38.87%)	113 (40.65%)	93 (25.34%)	
≥30%	313 (27.48%)	135 (27.33%)	72 (25.90%)	106 (28.88%)	
Knowledge of assessment method					
No	271 (24.82%)	108 (22.69%)	74 (26.91%)	89 (26.10%)	0.350
Yes	821 (75.18%)	368 (77.31%)	201 (73.09%)	252 (73.90%)	
Knowledge of performance					
No	225 (20.14%)	96 (19.67%)	64 (23.53%)	65 (18.21%)	0.242
Yes	892 (79.86%)	392 (80.33%)	208 (76.47%)	292 (81.79%)	
Value on income					
Low	568 (49.87%)	251 (50.81%)	122 (43.88%)	195 (53.13%)	0.057
High	571 (50.13%)	243 (49.19%)	156 (56.12%)	172 (46.87%)	
Satisfaction on income level					
Unsatisfied	883 (78.28%)	389 (79.39%)	222 (80.14%)	272 (75.35%)	0.253
Satisfied	245 (21.72%)	101 (20.61%)	55 (19.86%)	89 (24.65%)	
Satisfaction on PBS					
Unsatisfied	623 (56.13%)	293 (60.66%)	160 (59.48%)	170 (47.49%)	<0.001
Satisfied	487 (43.87%)	190 (39.34%)	109 (40.52%)	188 (52.51%)	

**Table 2:** The association between basic characteristics/PBS design and health workers’ satisfaction on payment methods (All sample).

Characteristics	β	OR	Std. Error	β 95% CI	*p*
Gender (Control: Male)					
Female	−0.26	0.77	0.18	(−0.62, 0.1)	0.16
Age (Control: <25)					
25–34	−0.52	0.59	0.38	(−1.27, 0.23)	0.17
35–44	−0.17	0.84	0.40	(−0.96, 0.62)	0.67
45–54	−0.06	0.95	0.45	(−0.94, 0.83)	0.90
≥55	0.24	1.27	0.61	(−0.95, 1.43)	0.69
Educational Background (Control: Bachelor’s and above)					
Junior college	−0.10	0.91	0.18	(−0.45, 0.26)	0.58
High school or below	−0.19	0.83	0.25	(−0.69, 0.31)	0.45
Professional status (Control: Senior/deputy senior)					
Intermediate	0.11	1.12	0.62	(−1.1, 1.32)	0.85
Primary	0.48	1.61	0.63	(−0.75, 1.71)	0.45
Lower than primary	0.56	1.74	0.67	(−0.76, 1.87)	0.41
Employment status (Control: Temporary)					
Permanent	0.34	1.41	0.23	(−0.1, 0.79)	0.13
Work role (Control: Doctor)					
Nurse	0.57	1.76	0.22	(0.13, 1.01)	0.01
Public health workers	0.66	1.94	0.21	(0.25, 1.07)	0.00
Percentage of floating income	−2.82	0.06	1.06	(−4.9, −0.75)	0.01
Knowledge on assessment method (Control: No)					
Yes	0.89	2.44	0.20	(0.5, 1.28)	<0.001
Knowledge on performance (Control: No)					
Yes	1.21	3.34	0.25	(0.72, 1.69)	<0.001
Value on income (Control: Low)					
High	0.39	1.48	0.26	(−0.12, 0.9)	0.13

**Table 3:** The association between PBS design and satisfaction on payment methods for health workers with different value on financial income.

Satisfaction with Payment Methods	High Value on Financial Income *n* = 737				Low Value on Financial Income *n* = 287			
	Satisfied (%)	Dissatisfied (%)	*p*	β 95%CI	Satisfied (%)	Dissatisfied (%)	*p*	β 95%CI
Gender								
Male	41.21	58.79			53.81	46.19		
Female	37.04	62.96	0.86	−0.04 (−0.46, 0.38)	47.83	52.17	0.01	−1.03 (−1.84, −0.22)
Age								
<25	34.62	65.38			67.86	32.14		
25–34	42.53	57.47	0.54	−0.28 (−1.17, 0.62)	49.66	50.34	0.07	−1.43 (−2.96, 0.1)
35–44	35.58	64.42	0.97	0.02 (−0.92, 0.96)	46.67	53.33	0.33	−0.79 (−2.39, 0.8)
45–54	37.33	62.67	0.79	−0.14 (−1.2, 0.92)	54.55	45.45	0.73	0.32 (−1.5, 2.13)
≥55	42.11	57.89	0.44	0.53 (−0.82, 1.88)	55.17	44.83	0.45	−1.19 (−4.24, 1.87)
Educational Background								
Bachelor’s and above	39.25	60.75			48.83	51.17		
Junior college	37.39	62.61	0.71	−0.08 (−0.5, 0.34)	48.28	51.72	0.53	−0.24 (−1, 0.51)
High school or below	38.18	61.82	0.69	−0.12 (−0.69, 0.46)	56.76	43.24	0.29	−0.58 (−1.65, 0.5)
Professional status								
Senior/deputy senior	30.00	70.00			62.50	37.50		
Intermediate	33.68	66.32	0.61	0.39 (−1.09, 1.87)	48.28	51.72	0.64	−0.68 (−3.53, 2.18)
Primary	38.87	61.13	0.47	0.56 (−0.95, 2.07)	47.52	52.48	0.84	0.30 (−2.6, 3.21)
Lower than primary	45.61	54.39	0.44	0.63 (−0.96, 2.23)	59.18	40.82	0.76	0.48 (−2.62, 3.58)
Employment status								
Permanent	36.08	63.92	0.29	0.27 (−0.23, 0.77)	47.06	52.94	0.18	0.73 (−0.33, 1.78)
Temporary	44.30	55.70			62.28	37.72		
Work role								
Doctor	36.93	63.07			41.74	58.26		
Nurse	34.21	65.79	0.17	0.35 (−0.15, 0.86)	48.72	51.28	<0.001	1.48 (0.49, 2.46)
Public health workers	43.11	56.89	0.03	0.54 (0.05, 1.02)	60.73	39.27	0.01	1.17 (0.29, 2.04)
Percentage of floating income	--	--	0.05	−2.36 (−4.73, 0.01)	--	--	0.04	−5.00 (−9.64, −0.36)
Knowledge of assessment method								
No	14.01	85.99			32.73	67.27		
Yes	46.32	53.68	<0.001	1.01 (0.56, 1.46)	53.50	46.50	0.15	0.65 (−0.23, 1.54)
Knowledge of performance								
No	11.20	88.80			17.00	83.00		
Yes	46.51	53.49	<0.001	1.22 (0.66, 1.77)	57.24	42.76	0.15	0.93 (−0.16, 2.01)

## Data Availability

Data available on request due to restrictions privacy. The data presented in this study are available on request from the corresponding author.
